# Derivation of functional early gestation decidual natural killer cell subtypes from induced pluripotent stem cells

**DOI:** 10.1101/2025.11.03.685424

**Published:** 2025-11-05

**Authors:** Virginia Chu Cheung, Carly DaCosta, Jennifer Jaimez, Harneet Arora, Christine Caron, Jaroslav Slamecka, Manuel Fierro, Morgan Meads, Kathleen Fisch, Robert E. Morey, Devika Pant, Dan S. Kaufman, Mariko Horii, Jack D. Bui, Mana M. Parast

**Affiliations:** 1 Department of Pathology, School of Medicine, University of California San Diego, La Jolla, CA,92093, USA.; 2 Sanford Consortium for Regenerative Medicine; 3 Center for Perinatal Discovery, University of California San Diego, La Jolla, CA, 92093, USA.; 4 Department of Obstetrics, Gynecology, and Reproductive Sciences, School of Medicine, University of California San Diego, La Jolla, CA, 92093, USA.; 5 Department of Medicine, School of Medicine, University of California San Diego, La Jolla, CA, 92093, USA.

## Abstract

Abnormal decidual natural killer cell (dNK) function is linked to pregnancy complications occurring in both early and late stages of gestation, including recurrent pregnancy loss, preeclampsia, and preterm birth. Exploration of dNK heterogeneity as it relates to function is an active area of research; however, most of this work has focused on early gestation. We applied flow cytometric and transcriptomic single cell definitions of dNK subtypes to dNK at the chorioamniotic membranes (CAM) and basal plate (BP) of the placenta at term. We found decreased low-effector-function dNK1 and increased high-effector-function dNK3 abundance at term. In comparison to BP-dNK, CAM-dNK had greater abundance of moderate-effector-function dNK2 and lower expression of inhibitory CD9. We applied this knowledge to establish a protocol for differentiation of induced pluripotent stem cells (iPSC) into CD45^+^CD56^bright^CD16^−^, functional dNK-like, applying TGFβ to enrich for dNK2 – the most abundant dNK subtype at first trimester–while inducing expression of dNK markers, CD9 and CD103. We analyzed the secretomes of first trimester dNK, term BP-dNK and CAM-dNK, and peripheral blood NK cells to identify proteomic profiles for each. Finally, we analyzed the secretome of TGFβ-treated iPSC-dNK and found an enrichment in first trimester dNK-specific proteins. We identify changes in dNK function across gestation and placental region and suggests that these changes can be explained by shifts in dNK subtypes, which we specifically and reproducibly derive from iPSC, providing a new model for these cells and laying the foundation for cell-based therapies of reproductive diseases.

## Graphical Abstract

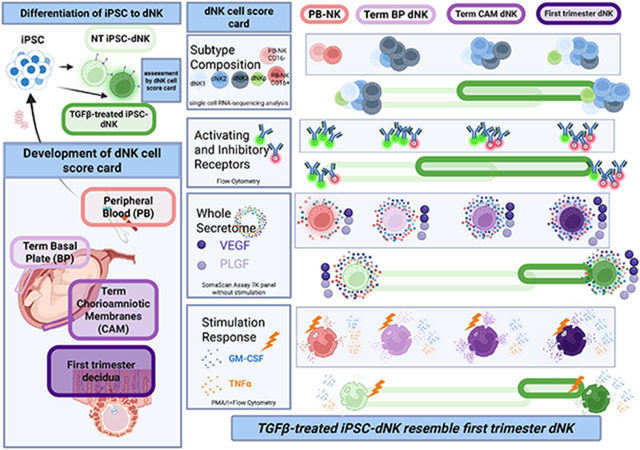

## Introduction:

A successful pregnancy requires maternal tolerance of the semi-allogeneic fetal-derived placenta.^1^ Prior to fertilization and embryo implantation, the uterine lining undergoes decidualization, a morphologic and functional transformation, leading to a tissue that is poised for both tolerance of fetal-derived antigens, and recognition and clearance of potential pathogens.^1,2^ This process involves infiltration with a vast array of maternal leukocytes, including natural killer (NK) cells.^3^ Decidual NK cells (dNK) are a type of tissue resident NK cells (trNK) found in the uterine lining. dNK cells function to support implantation and promote angiogenesis by crosstalk with maternal decidualized endometrial stromal fibroblasts (dESF) and fetal-derived extravillous trophoblasts (EVT) in the placenta.^4,5^ As gestation progresses, the maternal decidua contributes to the basal plate (BP, also the decidua basalis) and chorioamniotic membranes (CAM, also the decidua parietalis). dNK at the BP regulate EVT invasion and establish maternal blood flow to the feto-placental unit.^5^ Meanwhile, CAM functions as a protective barrier surrounding the fetus throughout much of gestation. At term, CAM is remodeled and ruptures, partly due to a shift in dNK function, from tolerance-inducing in early gestation to proinflammatory in late gestation.^6^ Abnormal dNK function has been associated with multiple pregnancy complications including recurrent spontaneous pregnancy loss, preeclampsia, as well as inflammation-induced spontaneous preterm birth.^7-10^

dNK at the maternal-fetal interface have cell surface marker expression and function distinct from peripheral blood NK cells (PB-NK). dNK in nonpregnant and first trimester decidua are CD56^bright^CD16^−^, and uniformly co-express CD9, unlike CD56^dim^CD16^+^CD9^−^ PB-NK.^11,12^ In the NK-92 cell line, acquisition of CD9 results in increased production of proangiogenic IL-8, reduced production of proinflammatory TNFα and IFNγ, and reduced cytotoxicity against target cells.^13^ However, dNK at the maternal-fetal interface are not the same throughout gestation. Inhibitory Killer Cell Immunoglobulin-Like Receptors KIR2DL1 and KIR2DL2/3 are reduced in both BP- and CAM-dNK cells at term compared to first trimester dNK.^16,17^ At baseline, dNK and PB-NK express cytolytic molecules (PFN, GZMB, GNLY); however, PB-NK have higher PFN and GZMB and first trimester dNK (but not term dNK) have higher GNLY and 9kD GNLY.^16^ Unstimulated, first trimester dNK, but not PB-NK, secrete proangiogenic cytokines and growth factors (IL-6, VEGF, PLGF). ^14,15^ These data are supportive of a role for dNK in vascular remodeling in early gestation. The function of unstimulated term dNK are less well understood as term dNK have never been evaluated in this capacity.

Nevertheless, it is known that PB-NK, first trimester dNK, and term dNK cells have distinct activation responses. PB-NK induce higher levels of CD107a and cytokines (TNFα, IFNγ, GM-CSF), compared to first trimester dNK, in response to phorbol- 12-myristate-13-acetate and ionomycin (PMA/I) stimulation.^14,16^ Given the same stimulation, term dNK (BP and CAM) respond with high CD107a, similar to PB-NK, but modest pro-inflammatory cytokine response (TNFα, IFNγ), similar to first trimester dNK. Although certain activation receptors (*CD69*, *NKp80*, *NKp44*) are expressed at the RNA level in CAM-dNK, but not in BP-dNK, functional and protein expression differences have not yet been identified.^16,18-20^ dNK function and functional receptor expression are distinct between first trimester and term (including between BP and CAM)- however, the basis for this functional change remains unclear.

Recent single-cell analyses have pointed to first trimester dNK’s transcriptional and functional heterogeneity, identifying 4 subtypes, dNK1, dNK2, dNK3 and dNKp.^21,22^ dNK1 express CD39 (encoded by *ENTPD1*), an ectonucleotidase expressed by trNK found in tonsil, liver, and first trimester decidua.^12^ CD39 is an inhibitory receptor, as mice deficient in this gene have increased NK cell numbers and NK cell effector functions.^23^ dNK2 and dNK3 express CD18 (encoded by *ITGB2*), an integrin that enhances NK cell cytotoxicity.^24^ dNK3 also express CD103 (encoded by *ITGAE*), a tissue residency marker for lung NK cells, as well as T cells in multiple tissue types.^25,26^ Expression of CD103 by dNK is associated with higher cytokine (GM-CSF, XCL1) and CD107a expression, after PMA/I stimulation.^22^ Indeed, following stimulation, dNK3 have the highest GM-CSF followed by dNK2, then dNK1 with low levels similar to PB-NK.^22^ Importantly, dNK subtypes differentially express receptors and ligands (*CCL5*, *LILRB1*, *CSF1*) used for interaction with placental EVT and other cell types at the maternal-fetal interface – suggesting distinct roles in placentation.^21^ However, it is not known whether these subtypes persist or change throughout gestation at different locations within the maternal-placental interface.

Due to lack of access to decidual tissues in an ongoing pregnancy, studies typically rely on model systems. Mouse placentation is distinct from human at structural, molecular, and cell-cell interaction levels.^27^ Importantly, human and mouse placental cells at the maternal-placental interface, do not have the same expression of key receptor/ligand pairs involved in dNK crosstalk.^28^ Similarly, PB-NK and term dNK significantly differ from their first trimester counterparts, both in their immunophenotype and functional capacity.^3,16,29-31^ While attempts have been made to generate dNK from PB-NK, primary NK cells are difficult to reliably expand in culture – limiting their capacity for mechanistic studies.^32^ This necessitates novel models for the study of human dNK and their integral interactions with maternal and placental cells.

Over the past two decades, generation of patient-specific induced pluripotent stem cells (iPSC) from somatic cells has revolutionized the modeling of numerous human diseases.^33^ We and others have shown that preeclampsia (PE)-associated cellular abnormalities can be modeled using iPSC. This includes abnormal differentiation and function of trophoblast, derived from iPSC, obtained by reprogramming umbilical cord cells from PE-affected placentas,^34-36^ as well as abnormal function of endothelial cells, derived from iPSC, obtained by reprogramming PBMCs from patients with PE.^37^ Currently, there are protocols to routinely generate mature, cytotoxic CD56^+^CD16^+^ NK cells from iPSC. These iPSC-NK are transcriptionally similar to umbilical cord and PB-NK, and kill tumor cells *in vitro* and *in vivo.*^38-40^ Several clinical trials have now demonstrated that engineered iPSC-derived NK are both safe and effective in treatment of refractory malignancies.^33,41^ However, no protocol exists to generate dNK from iPSC.

Studies in mice and human suggest a mechanism of dNK emergence is through PB-NK infiltration and exposure to the decidual niche,^42,43^ where the oxygen tension throughout gestation is 2-8% O_2_.^44^ In fact, IL-15 and exposure to low oxygen tension induce a dNK-like phenotype in PB-NK.^32,45^ Trophoblast-derived SDF-1 (*CXCL12*) stimulate PB-NK migration into decidua, with PB-NK coculture with EVT promoting conversion to a dNK-like phenotype.^42,46^ Furthermore, treatment of PB-NK with TGFβ1, a growth factor secreted by maternal dESF and placental EVT, in combination with hypoxia, promote a dNK-like phenotype, including induction of CD9, reduced cytotoxicity, and increased VEGF secretion.^25,32^ In this study, we characterize dNK heterogeneity and function at first trimester and different regions of the term placenta, and apply this improved understanding of dNK surface protein expression, gene expression, and protein secretion to establish a differentiation protocol to derive functional dNK from iPSC.

## Results:

### Defining primary dNK subtypes by scRNA-seq and flow cytometry across gestation

**Figure 1.**
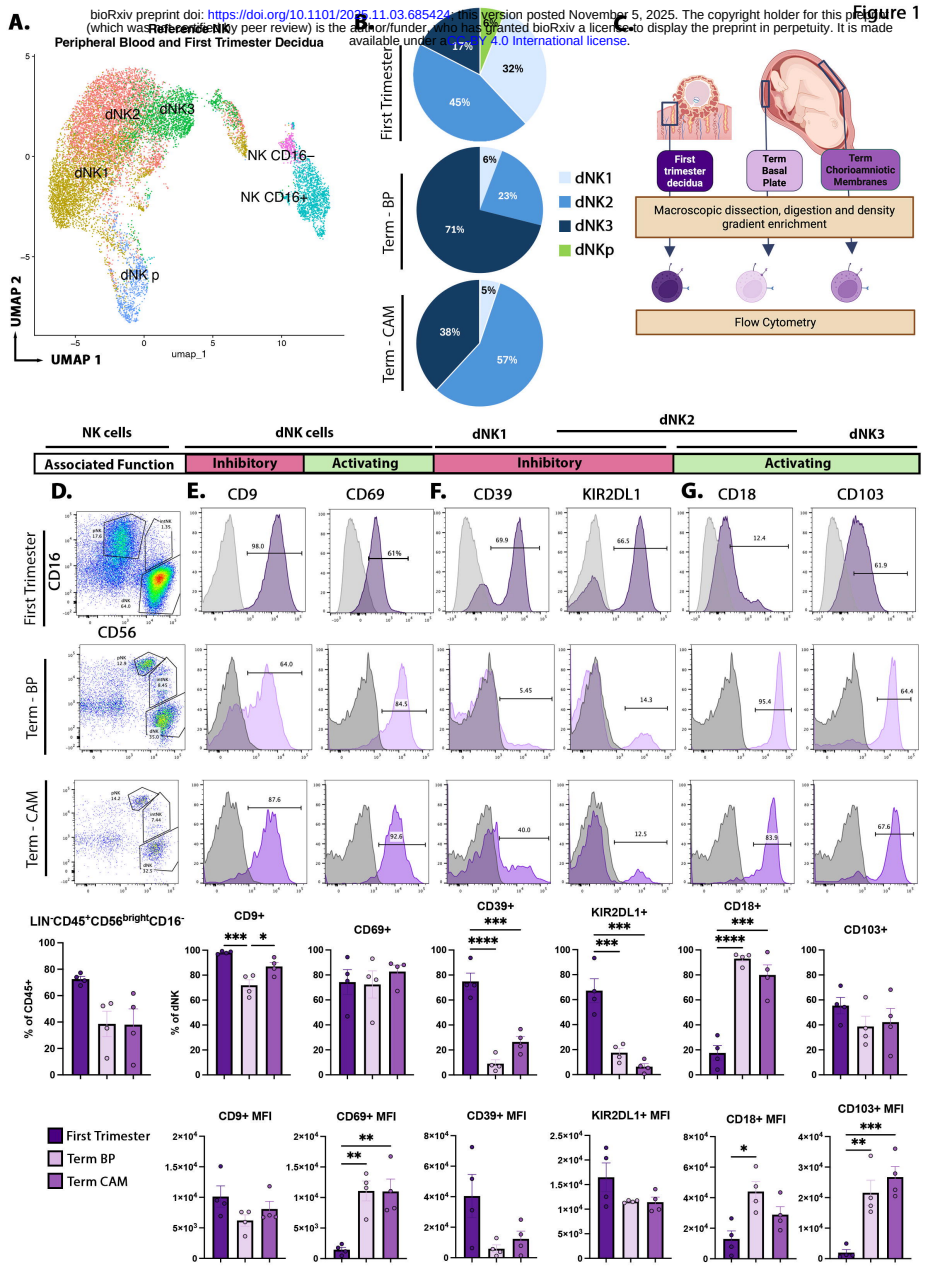
Term dNK cells show enrichment in activated dNK subtypes. **A)** UMAP showing NK cell reference from Vento-Tormo, 2018^21^ containing dNK and PB-NK subtypes. **B)** Pie chart showing dNK subtype composition in decidua from first trimester, term basal plate (BP), and term chorioamniotic membrane (CAM), as identified by reference mapping term scRNA-seq data from Pique-Regi et al., 2019^46^ to **(A)** (data from Pique-Regi reanalyzed with permission). **C)** Schematic of primary cell samples used for flow cytometric analysis. **D-G)** Representative FACS plots and quantification of NK cell markers (CD56 and CD16), in first trimester decidua, term BP, and term CAM after gating on Lin^−^CD45^+^CD56^bright^CD16^−^ cells **(D)**, dNK markers (CD9 and CD69) **(E)**, inhibitory receptors (CD39 and KIR2DL1) **(F)**, and activating receptors (CD18 and CD103) **(G)**. For **D** through **G**, data are represented as mean +/− standard error, n = 8 individual patients (4 first trimester donors and 4 term placenta donors; BP and CAM were dissected and processed separately from the same term placenta). Statistical testing was Ordinary one-way ANOVA with multiple comparisons *p<0.05, **p<0.01, ***p<0.001, ****p<0.0001. Alt text: UMAP showing NK cell heterogeneity. Pie charts showing dNK subtype composition across gestation. Histograms and bar graph quantification of cell surface marker expression on first trimester, Term BP, and Term CAM dNK cells.

We set out to determine if the first trimester dNK subtypes (dNK1, dNK2, dNK3, and dNKp) could also be identified in term decidua. ^21,22^ We reanalyzed previously-published scRNA-seq datasets from blood and first trimester decidua (Vento-Tormo, 2018) as well as term placental and decidual tissues (Pique-Regi, 2019).^21,46^ We utilized all cells in the Vento-Tormo dataset, and verified NK cells by expression of NK-associated genes (Supplementary Figure 1A, B). We extracted all the NK cells (peripheral blood-derived, PB-NK; and decidual-derived NK, dNK) from the Vento-Tormo dataset (Figure 1A), to use as a reference. We compared gene expression between these reference NK cells, in the absence of non-NK cell types, to identify new NK cell subtype-specific marker genes for dNK1, dNK2, dNK3, PB-NK CD16^+^, and PB-NK CD16^−^ (Figure 1A, Supplementary Figure 1C, Table 1, Supplementary File 1).

In the Pique-Regi dataset, three compartments in the term placenta (BP; CAM; and placental villus, PV) were annotated using sample-associated meta data (Supplementary Figure 1D,E). We verified NK cells by expression of NK-associated genes (Supplementary Figure 1F). We extracted all NK cells from the Pique-Regi dataset and mapped them to our reference NK cells (from the Vento-Tormo dataset).^21^ All dNK subtypes found in first trimester could be identified at term, with dNK2 making up the majority of the dNK in first trimester decidua (45%) and term CAM (57%) and dNK3 making up the majority in term BP (71%). (Figure 1B). Meanwhile, dNK1 made up only 6% of BP-dNK and 5% of CAM-dNK, while ≥0.1% of all term dNK mapped to dNKp (Figure 1B). An abundance of cells expressing dNK3 marker, *CCL5*, with smaller populations of cells expressing the dNK2 marker, *ZNF683* (also HOBIT), and dNK1 marker, *SPINK2*, and only rare cells expressing dNKp marker *MKI67* (Supplementary Figure 1C,G,H, Table 1) in term NK cells. We found there were more dNK2 marker, *ZNF683*^+^, NK cells in CAM and more dNK3 marker*, CCL5*^+^, NK cells in BP (Supplementary Figure 1I). This agrees with the reference mapping results, which identify more dNK2 in CAM and more dNK3 in BP (Figure 1B). The Pique-Regi dataset contains cells from placentas delivered at term, with and without labor, and preterm with labor. Across all three labor groups, dNK3 were increased in both compartments relative to first trimester, with dNK3 dominant in BP, and dNK2 the dominant in CAM (Supplementary Figure 1J.).

We next characterized dNK in early first trimester decidua and term placenta, the latter including decidua from both BP and CAM, by flow cytometry (Figure 1C). We confirmed that dNK are CD45^+^CD56^bright^CD16^−^ and comprise 40-70% of CD45^+^ cells in first trimester and term decidual tissues (Figure 1D). dNK-associated CD9 and trNK-associated CD69 were expressed by a large proportion of dNK throughout gestation (Figure 1E). ^21,22^ Quantification by Median Fluorescent Intensity (MFI) showed a significant increase in CD69 in term dNK in both compartments (Figure 1E).

We next evaluated dNK subtype-associated markers. dNK1 marker CD39 (*ENTPD1*) was expressed by an average of 74.9% of dNK in first trimester but was reduced over 8-fold (to 9.0% average) in term BP and almost 3-fold (to 26.5% average) in term CAM (Figure 1F). ^21,22^ Similarly, KIR2DL1, a marker of both dNK1 and dNK2, was expressed by an average of 67.2% of first trimester dNK, but only 17.5% and 6.4% of term BP and CAM, respectively (Figure 1F). ^21,22^ In contrast, CD18 (*ITGB2*), a marker of both dNK2 and dNK3 was ~5-fold higher in term BP (93.0% average) and term CAM (80.0% average), compared to first trimester (17.6% average) (Figure 1G). dNK3 marker CD103 (*ITGAE*) was expressed by an average of ~55%, ~39%, and 42% of the dNK in early gestation decidua, term BP, and term CAM, respectively (Figure 1G). ^21,22^ However, like CD18, CD103 MFI was upregulated at term, increased by ~11-fold and 13.5-fold in term BP and CAM, respectively (Figure 1G). This is consistent with scRNA-seq results, which show that dNK1 are more abundant in first trimester decidua, while dNK3 are relatively more abundant at term, in both BP and CAM. Flow cytometry of dNK2-associated KIR2DL1 and CD18 were incongruous; however, scRNA-seq analysis, which uses more markers, identified dNK2 as the most abundant cell type in first trimester decidua (Figure 1B).

Term BP- and CAM-dNK have higher activation responses than first trimester dNK.^16^ When first trimester dNK is sub-stratified by subtype, dNK3 has the highest effector response, followed by dNK2, and then dNK1.^22^ In agreement with the literature, we found markers associated with decreased effector function (CD9, KIR2DL1 and CD39) to be higher in first trimester dNK, while activating receptors (CD18, CD103, and CD69) was increased at term, in both BP and CAM (Figure 1E-G). Interestingly, CD39, KIR2DL1, and CD18 was dramatically changed between first trimester and term. CD9, CD69, and CD103 was maintained by a more constant proportion of dNK, although levels of CD69 and CD103 were higher in term, in comparison to first trimester. Together, these data suggest that functional changes in dNK across gestation may be driven by a shift in dNK subtypes, with decreased dNK1 (low effector function, high levels of inhibitory molecules) and increased dNK3 (high effector function, high levels of activating molecules) as gestation progresses toward term.

### iPSC-NK acquire dNK markers in response to TGFβ treatment

**Figure 2.**
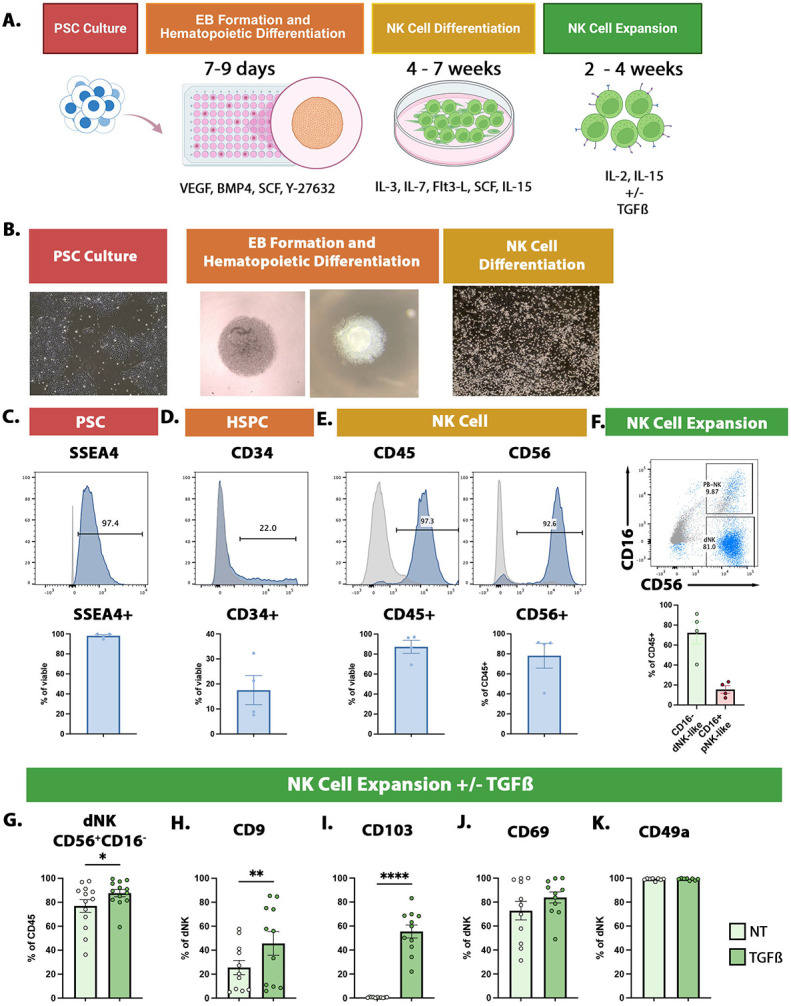
TGFβ supplementation increases dNK cell marker expression in iPSC-derived NK cells. **A)** Schematic of the entire differentiation protocol. **B)** Representative phase images of cells throughout the differentiation process. **C-E)** Representative FACS plot and quantification of expression over isotype control. Pluripotency marker SSEA4 staining in iPSC, before starting the differentiation **(C)**, hematopoietic stem progenitor cell (HSPC) marker CD34 staining after EB formation, before plating for NK cell differentiation **(D)**, and immune cell marker CD45 and NK cell marker CD56 staining after NK cell differentiation, before plating for expansion **(E)**. **F)** Representative FACS plot of CD56 and PB-NK cell marker CD16 co-staining after at least 2 weeks of expansion in IL-2 and IL-15. dNK cell-like (CD45^+^CD56^+^CD16^−^) and PB-NK cell-like (CD45^+^CD56^+^CD16^+^) populations are quantified. **G)** Quantification of dNK cell-like (CD45^+^CD56^+^CD16^−^) population in No Treatment (NT, no TGFβ) and TGFβ treatment conditions. **H-K)** Quantification of flow cytometric staining of dNK cell associated markers, CD9 **(H)**, CD103 **(I)**, CD69 **(J)**, and CD49a **(K)**, in iPSC-dNK cells at the end of the expansion phase. For **C** through **K**, data are represented as mean +/− standard error. For **C** through **F**, n = 4 PSC lines; H9 hESC line and MB3140, MB3144, LD2809 iPSC lines. For **G** through **K,** n = 11 independent experiments using MB3140, MB3144, LD2809 iPSC lines, Statistical testing was using Ordinary one-way ANOVA with multiple comparisons, *p<0.05, **p<0.01, ***p<0.001, ****p<0.0001. Alt text: Schematic of differentiation protocol. Bright phase images of cells during differentiation. Histograms and bar graphs quantifying expression of markers during key phases of differentiation. Scatter plot and bar graphs showing dNK-like and PB-NK-like cells. Bar graphs showing increased dNK marker expression in iPSC-dNK after TGFbeta treatment.

We next set out to establish an *in vitro* model of dNK, by differentiating hPSC. We used a protocol, modified from Zhu et al., 2019,^47^ to differentiate the hESC line H9/WA09, and 3 different iPSC lines, generated and validated in our own lab (Supplementary Figure 2A-E), into NK-like cells. At the start of this protocol (Figure 2A), hematopoietic stem/progenitor cells were generated from highly pure, SSEA4^+^ hPSC (Figure 2B, C), using a spin embryoid body (EBs) method. EBs containing CD34^+^ cells (Figure 2B, D) were plated for NK differentiation. After 28 days, round cells emerged in suspension. 87.3% of these cells were CD45^+^, of which 78.2% were also CD56^+^ (Figure 2B, E). To enrich for NK, these cells were collected for expansion. Instead of coculture with artificial antigen presenting cells as previously published,^47^ we cultured the cells in a commercially available media (see Methods) supplemented with IL-2 and IL-15 for this expansion phase. After 2 weeks of expansion, 87.8% of CD45^+^ cells were also CD56^+^, with 15.6% coexpressing CD16 (CD56^+^CD16^+^, PB-NK-like), and 72.2% lacking CD16 (CD56^+^CD16^−^, dNK-like) (Figure 2F).

We hypothesized conditions mimicking the decidual niche would enhance/enrich a dNK-like phenotype. We reclustered the NK cells from the Vento-Tormo dataset broadly into two groups: dNK (comprised of dNK1, dNK2, dNK3, and dNKp), or PB-NK (comprised of NK CD16^+^ and NK CD16^−^) (Supplementary Figure 3A) and compared the two to identify an enrichment in TGFβ signaling and hypoxia pathways in dNK (Supplementary Figure 3B). Additionally, TGFβ and CXCL12 promote dNK-like phenotype when applied to PB-NK.^25,42^ We tested TGFβ and CXCL12 under 5% or 21% O_2_, during the expansion phase of our protocol, and found TGFβ, but not CXCL12 or 5% O_2_, slightly increased the proportion of CD56^+^CD16^−^, decreased CD18, and significantly increased dNK markers CD9 (2-fold) and CD103 (~90-fold) (Figure 2G, H, I, Supplementary Figure 3C, D). TGFβ had no effect on CD69 and dNK cell-associated CD49a (Figure 2J,K).

Together, these data find that we can derive dNK-like cells from iPSC, and that TGFβ pushes the cells further toward this phenotype.

### iPSC-derived NK are transcriptionally similar to dNK, not PB-NK, and TGFβ induces dNK2

**Figure 3.**
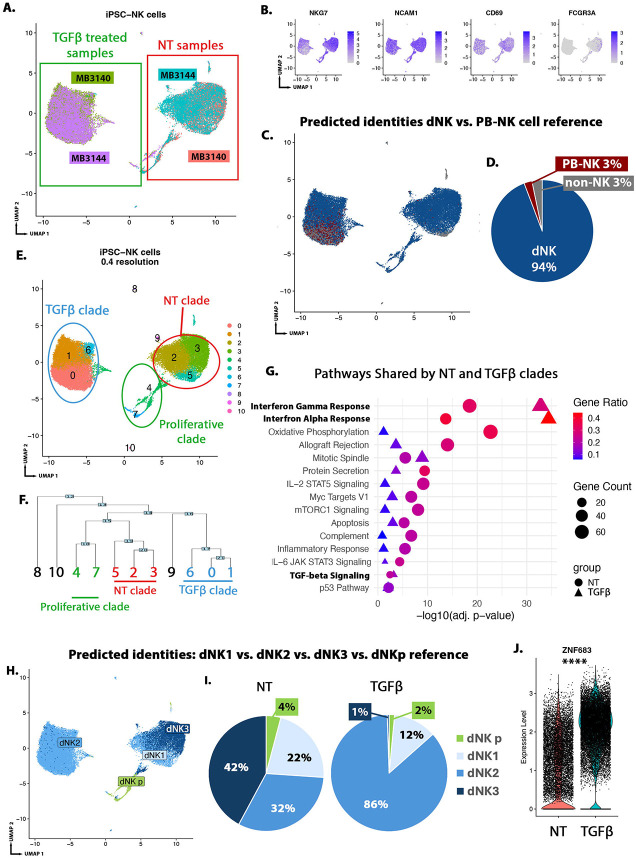
iPSC-derived NK cells transcriptionally resemble dNK cells, with TGFβ promoting a dNK2 phenotype. **A)** UMAP showing no treatment (NT, no TGFβ) and TGFβ -treated iPSC-NK cells derived from MB3140 and MB3144 iPSC lines. Unsupervised clustering resulted in separation by treatment group with TGFβ-treated cells on the left and NT on the right. **B)** FeaturePlots showing expression of dNK marker genes (*NKG7*, *NCAM1*, *CD69*) and absence of PB-NK marker gene (*FCGR3A*), which encodes CD16. **C)** UMAP showing predicted identities for iPSC-NK, using all cells from Vento-Tormo et al., 2018^21^ as reference. **D)** Pie chart quantification of data in **(C)**, the predicted identities of iPSC-NK, using data from Vento-Tormo et al., 2018^21^ as reference. **E)** UMAP showing clustering of iPSC-NK cells at 0.4 resolution. **F)** Dendrogram showing phylogenetic analysis of aggregate gene expression of iPSC-NK cell clusters. TGFβ clade in blue, NT clade in red, Proliferative clade in green. **G)** Quantification of pathways significantly enriched in both NT and TGFβ clades using MSigDB Hallmark 2020 pathway analysis. There are two values per pathway, one each from NT clade-enriched DEGs (circles) and TGFβ clade-enriched DEGs (triangles). **H)** UMAP showing all predicted identities for iPSC-NK cells, using the dNK cell subtypes (dNK1, dNK2, dNK3, dNKp) from Vento-Tormo, 2018^21^ as reference. **I)** Pie chart quantification of data in **(H)**, showing all predicted identities for iPSC-NK cells by reference mapping to dNK cell subtypes (dNK1, dNK2, dNK3, dNKp) from Vento-Tormo, 2018.^21^ **J)** *ZNF683*, dNK2 marker gene, has significantly higher expression in TGFβ-treated iPSC-NK cells in comparison to NT iPSC-NK. ****p < 0.0001 by Wilcox Rank Sum test. Alt text: UMAPs showing NK cell marker expression by iPSC-NK. UMAPs and Pie chart showing dNK cell identify of 94% of iPSC-NK. UMAP and dendrogram showing clustering of iPSC-NK by treatment group. Graph showing gene enrichment in dNK-associated pathways by iPSC-NK, with further enrichment upon TGFbeta treatment. UMAP and pie chart showing iPSC-NK resemble all dNK subtypes with increased proportion of dNK2 upon TGFbeta treatment. Violin Plot showing higher expression of dNK2-marker gene ZNF683 in TGFbeta-treated iPSC-NK

We further characterized our iPSC-NK, with and without TGFβ, using scRNA-seq (Figure 3A). We found that iPSC-NK expressed dNK marker genes (*NCAM1*, *ITGA1*, *CD69*) and no *FCGR3A* (PB-NK-associated CD16) (Figure 3B). We again used the full Vento-Tormo dataset as a reference, with NK cells subclustered broadly as dNK (comprised of dNK1, dNK2, dNK3, and dNKp), or PB-NK (comprised of NK CD16^+^ and NK CD16^−^) (Supplementary Figure 3A).^21^ We found 94% of iPSC-NK cells mapped to dNK (Figure 3C,D). iPSC-NK also expressed dNK (*SPINK2*, *ZNF683*, *CCL5*), but not PB-NK (*SELL*, *SPON2*) subtype markers (Supplementary Figure 4A). These data indicate that the iPSC-NK, regardless of TGFβ treatment, transcriptionally resemble dNK, not PB-NK.

We next investigated iPSC-NK heterogeneity using unsupervised and supervised analyses. Unsupervised clustering of iPSC-NK resulted in 11 clusters. Clusters 0-7 represent the majority (99%) of cells (Figure 3E; Supplementary Figure 4B). Hierarchical clustering identified clusters 0, 1, and 6 group into a clade, comprised of TGFβ-treated iPSC-NK (Figure 3F), expressing TGFβ-inducible genes, *JUN* and *SMAD7* (Supplementary Figure 4C). Clusters 2, 3, and 5 grouped into another clade, comprised of no treatment (NT) iPSC-NK (Figure 3F). Clusters 4 and 7 grouped into a third clade, comprised of G2M and S phase cells expressing proliferation genes (*MKI67*, *PCNA*). (Supplementary Figure 4D,E). Finally, clusters 8, 9, and 10, comprising a small minority (0.5%) of the cells, mapped primarily to non-NK populations (Figure 3C)

We identified marker genes for each cluster and continued with gene enrichment analysis of each of the major clades (Supplementary File 2). Both the NT and TGFβ clades were enriched for many pathways involved in NK cell function such as Protein Secretion, IL6 JAK/STAT3 signaling, and IL-2/STAT5 signaling (Supplementary File 3, Figure 3G). ^48-50^ We also found an enrichment for dNK-specific signaling pathways, Interferon Alpha and Gamma Response, and TGF-beta signaling (Figure 3G, Supplementary Figure 3B). Interestingly, all three of these dNK-specific pathways were more significantly enriched within the TGFβ clade (Figure 3G, triangle). As expected, DEGs in the proliferating clade were enriched for cell cycle control (Supplementary File 3). Notably, there enrichment for “Lymphoid cells of the Placenta” in DEGs from both the NT and TGFβ clades (Supplementary Tables 1-3), consistent with the dNK identity predicted by reference mapping, though pathway analysis identified differential activation of immune signaling pathways between these clades. This indicates that the iPSC-NK have dNK-associated gene expression that is further enriched by TGFβ.

To compare the heterogeneity of the iPSC-NK to that of dNK *in vivo,* we extracted the dNK subclusters (dNK1, dNK2, dNK3, dNKp),^21^ to use as a reference for iPSC-NK (Supplementary Figure 4F). We found the majority of cells (42%) in the NT clade mapped to dNK3, with the next largest population of cells (32%) mapping to dNK2, followed by dNK1 (22%). Examination of the TGFβ clade found 86% of cells mapping to dNK2, 12% to dNK1, and only 1% to dNK3 (Figure 3H,I, Supplementary Figure 4G). Further, the iPSC-NK expressed dNK, but not PB-NK, marker genes, and showed an enrichment of dNK2 marker gene *ZNF683* within the TGFβ-treated clade (Figure 3J, Supplementary Figure 4A).

In summary, scRNA-seq analysis confirmed the dNK identity of iPSC-derived NK cells. We identified heterogeneity within the iPSC-dNK, representing relevant dNK subtypes and that TGFβ further enriches for dNK signaling pathways and alters the proportion of dNK subtypes in favor of a dNK2.

### iPSC-NK functionally resemble dNK

With the dNK identity of the iPSC-derived NK cells established, we analyzed the function of these iPSC-dNK. dNK from early and late gestation express cytolytic proteins.^16^ We found that NT and TGFβ-treated iPSC-dNK expressed *GNLY*, *PRF1*, and *GZMB* (Supplementary Figure 5A) by scRNA-seq. We also found protein expression, albeit at low levels, of PFN (encoded by *PRF1*) and the first trimester dNK-specific 9kD GNLY isoform in NT and TGFβ-treated iPSC-dNK (Supplementary Figure 5B-C).^16^

**Figure 4.**
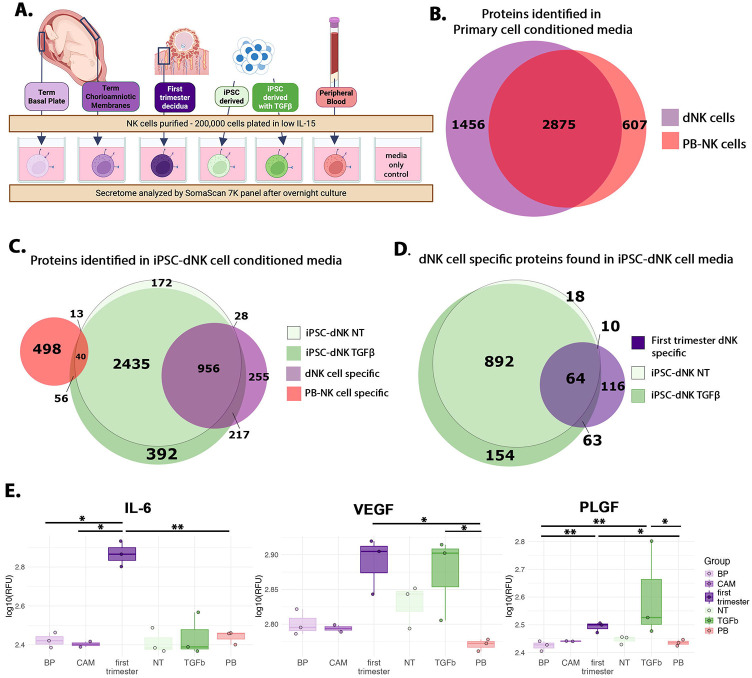
iPSC-derived dNK cells secrete proteins similar to primary dNK cells. **A)** Schematic of experimental design for aptamer-based proteomic profiling. **B)** Venn Diagram showing 2,875 proteins identified in conditioned media of first trimester, term BP-, and term CAM-dNK cells, as well as PB-NK cells; 1,456 proteins found only in primary dNK cell media (dNK cell specific); and 607 proteins found only in PB-NK cell media (PB-NK cell specific). **C)** Venn Diagram showing overlap of proteins identified in secretome of NT and TGFβ-treated iPSC-dNK cells with dNK cell-specific (1,456) and PB-NK cell-specific (607) proteins; more significant overlap was noted with the dNK cell-specific proteins. **D)** Venn Diagram showing overlap of proteins identified in secretome of NT vs. TGFβ-treated iPSC-dNK cells with first trimester dNK-specific (253) proteins; more significant overlap was noted with TGFβ-treated iPSC-dNK cell proteins. **E)** Box plots showing median expression as well as individual log_10_RFU values for each sample. Proteins known to be enriched in dNK (over PB-NK) cells are shown: IL-6 (seq.2573.20), VEGF (seq.2597.8), and PLGF (seq.2330.2). Dunn’s test for multiple comparisons is shown *p<0.05, **p<0.01. Alt text: Schematic illustrating all NK cells analyzed by apatmer-based proteomics profiling. Venn diagrams showing primary dNK secrete more proteins than PB-NK. Venn diagram showing iPSC-dNK secreted more dNK-specific proteins than PB-NK specific proteins. Venn diagram showing TGFbeta-treated iPSC-dNK express more first trimester dNK specific proteins than NT iPSC-dNK. Box and whisker plots showing higher levels of IL-6, VEGF, and PLGF proteins in first trimester dNK and TGFbeta-treated iPSC-dNK.

First trimester dNK, not PB-NK, express IL-6, VEGF, and PLGF in the absence of stimulation.^14,15^ However, it is not known if term or iPSC-dNK share this function. We purified NK cells from peripheral blood, first trimester decidua, and term BP and CAM, and compared them to dNK derived from iPSC with and without TGFβ per our protocol (Figure 4A). We applied aptamer-based proteomic profiling using the SomaScan Assay 7K panel (See Methods) to conditioned media and found 4,938 proteins expressed by primary cells at levels above media-only control, of which 1,456 were found in first trimester, BP, or CAM dNK, but not PB-NK (Figure 4B); of the latter, 570 proteins were shared by all 3 primary dNK (Supplementary Figure 6). We found iPSC-dNK secreted proteins had substantial overlap with those from primary dNK, and not PB-NK(Figure 4C). TGFβ, induced secretion of a markedly higher number of dNK-specific proteins, compared to NT iPSC-dNK (217 in TGFβ-treated, vs. only 28 in NT iPSC-NK) (Figure 4C).

TGFβ-treated iPSC-NK were enriched for dNK2, the most represented subtype in first trimester dNK (Figure 1B). We therefore asked if the secretory function of TGFβ-treated iPSC-dNK reflected that of first trimester dNK. Of the 253 first trimester dNK-specific proteins (Supplementary Figure 6), 127 were identified in TGFβ-treated iPSC-dNK secretome while only 74 were identified in NT iPSC-dNK (Figure 4D). IL-6, VEGF, and PLGF are secreted by first trimester dNK, not PB-NK.^14,15^ In agreement with this literature, we found levels of IL-6, VEGF, and PLGF were higher by 2.5-fold, 2.7-fold, or 2.1-fold, respectively, in first trimester dNK, compared to PB-NK, secretomes (Figure 4E). We also report, for the first time, that the high IL-6 and PLGF are in fact specific to first trimester dNK, as their levels are lower in BP-dNK and CAM-dNK, similar to PB-NK, secretomes. Interestingly, we found TGFβ-treated iPSC-dNK secrete higher VEGF and PLGF, similar to first trimester dNK (Figure 4E).

**Figure 5.**
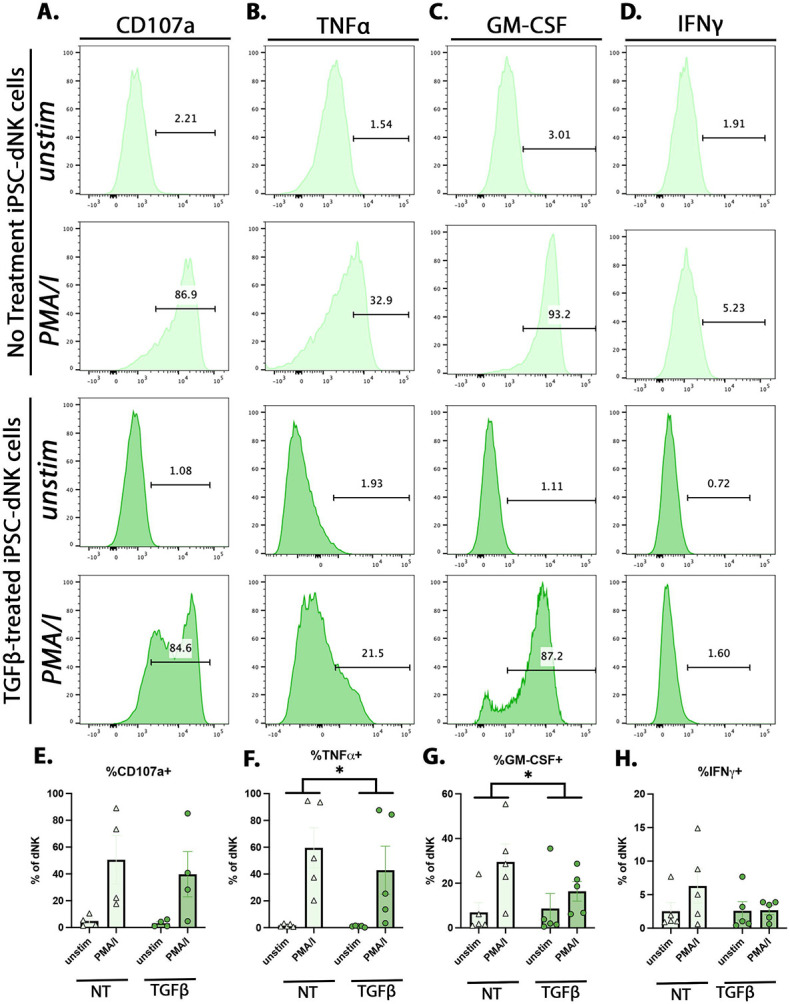
iPSC-derived dNK cells’ response to stimulation is modulated by TGFβ. Representative FACS plots for **A)** CD107a **B)** TNFα **C)** GM-CSF, and **D)** IFNγ of NT and TGFβ-treated iPSC-dNK in the presence or absence (unstim) of PMA/I treatment. Quantification of stimulation markers **E)** CD107a **F)** TNFα **G)** GM-CSF, and **H)** IFNγ, as a percent of expression of dNK cells, as measured by flow cytometry. Statistical testing was performed on PMA/I – unstim of n=4 independent experiments are iPSC-dNK derived from MB3140 and MB3144 iPSC lines. Data in **E** through **H** are represented as mean +/− standard error, by paired t-test *p<0.05. Alt text: Histograms and bar graph quantification showing iPSC-dNK increase CD107a, TNFalpha, GM-CSF, and IFNgamma in response to PMA/I stimulation. Statistical analysis shown on bar graphs show decreased TNFalpha and GM-CSF by TGFbeta-treated iPSC-dNK

Primary term dNK and PB-NK have similarly higher degranulation (CD107a), in comparison to first trimester dNK to PMA/I stimulation. However, stimulated first trimester and term dNK have lower production of cytokines (IFNγ, TNFα) than PB-NK.^16^ GM-CSF, however, has a reciprocal response, with higher induction in first trimester dNK than PB-NK. ^22^ Within first trimester dNK subtypes, in comparison to dNK2, dNK3 responsed with higher levels of CD107a, GM-CSF, and IFNγ.^22^ We found iPSC-dNK are responsive to PMA/I stimulation, inducing CD107a, IFNγ, TNFα, and GM-CSF, with decreased production of TNFα and GM-CSF by TGFβ-treated iPSC-dNK (Figure 5A-D). In summary, iPSC-dNK are functionally similar to primary dNK and TGFβ-treated iPSC-dNK have increased secretion of first trimester dNK-specific proteins and reduced stimulation response, supportive of a first trimester dNK (dNK2-dominant) phenotype.

## Discussion:

dNK are a key cell type during pregnancy, playing vital roles from implantation in early gestation, to continued regulation of EVT function at BP, and regulation of membrane rupture in CAM during labor. These various functions may be mediated by different dNK subtypes.^21^ We found dNK subtype compositions between first trimester decidua, term BP, and term CAM reflected this change in function. We also present novel analysis of NK cell function in our study, and report the complete secretome of first trimester, term BP, and term CAM dNK, as well as PB-NK. We used these primary cell data, in combination with published literature to develop a rigorous dNK scorecard to benchmark newly developed models. To this end, we report a robust, reproducible, and tunable protocol for induction of functional dNK-like cells from iPSC, as measured by cell surface markers, transcriptome, secretome, and functional activation.

In agreement with previous literature, we found term dNK have a more activated phenotype, compared to first trimester dNK. We identified a high proportion of high-effector function dNK3 at term, increased from first trimester, suggesting this increase in dNK3 is mediating the increased activating receptors, decreased inhibitory receptors, and increased activation observed in term dNK. However, while we found BP-dNK to have the greatest proportion of dNK3 based on scRNA-seq analysis, flow cytometry of CD18 (dNK2 and dNK3) and CD103 (dNK3) did not find these significantly increased, relative to first trimester or term-CAM dNK. This suggests that the transcriptomic and proteomic definitions of dNK subtypes are not in complete alignment, highlighting the need to develop a unified definition of dNK.

Studies of term dNK have shown no differences in protein expression or function based on localization in BP vs. CAM. However, term dNK from the two compartments do show gene expression differences.^16^ scRNA-seq analyses have pointed to a relative increase in dNK in CAM, but not BP, in placentas of patients with preterm labor.^46^ Further, labor-associated differential interactions were identified for BP-dNK and CAM-dNK.^51^ We reanalyzed the Pique-Regi, 2019 scRNA-seq dataset and identified similar differences between BP- and CAM-dNK. ^46^ Notably, we found that ~50% of the dNK in CAM are dNK2, similar to first trimester, while the dNK2 population is reduced (to 23%) in BP. In combination with the greater proportion of dNK3 in term BP, our data suggest that, compared to first trimester and term CAM, term BP dNK have a more activated phenotype and that could be driven by increased dNK3, over dNK2. dNK3 dominance over dNK2 in BP and dNK2 over dNK3 in CAM was consistent between term with and without labor (see Supplementary Figure 1J). Interestingly, we observed increased dNK3 in CAM in placentas with labor (term or preterm) but decreased dNK3 in BP of placentas from patients with preterm labor. Due to the small sample size (n=3 per labor group) we can only conclude that these observations warrant follow up studies on dNK subtypes in the context of labor, particularly preterm labor.

We found the proportion of dNK2 by scRNA-seq and proportion of CD9^+^CD56^bright^CD16^−^ dNK by flow cytometry were similar between first trimester decidua and term CAM; meanwhile, term BP showed decreased dNK2 based on scRNA-seq and decreased CD9^+^CD56^bright^CD16^−^ by flow cytometry. While the frequencies of CD9^+^ dNK (~90%) and dNK2 (~50%) are not the same within a single tissue, these data suggest CD9 could be used, in combination with other markers, to identify dNK2. We add further support for this relationship using our newly developed differentiation protocol for iPSC-dNK. TGFβ treatment resulted in selection against dNK3, in favor of dNK2, subtype, based on transcriptome, increased CD9 by flow cytometry, as well as reduced activation by functional analysis. These primary and iPSC-derived data suggest that CD9 may be a novel marker of a dNK2 transcriptional phenotype however additional experiments would be required to confirm this. Our study begins to bring the transcriptomic, flow cytometric, and functional definitions of dNK together, using primary and iPSC-derived dNK, and we expect our iPSC-derived model will accelerate research in this space.

Our study is the first application of untargeted aptamer-based proteomic profiling of secretome from primary NK cells, including first trimester and term dNK and PB-NK. Applying this same method to iPSC-dNK, we compared them, functionally, to primary dNK. Of the more than 4,000 proteins identified in the secretomes of primary NK cells, we found approximately 1/3 to be dNK-specific. Our study includes analysis of primary dNK from different gestational ages and decidual locations. We find similarities across gestation – with significant overlap between first trimester and term BP dNK - as well gestational age-related changes, such as reduced proangiogenic proteins at term. Notably, these proangiogenic proteins were upregulated in TGFβ-treated iPSC-dNK, suggesting these could be a signature of dNK2, which are the most abundant subtype in first trimester and induced by TGFβ. These cytokines can promote immune cell recruitment and expansion, which we can now model using our iPSC-dNK, but it remains to be determined if this function is specific to dNK2. Future studies are needed to define specific secretome signatures for each dNK subtype.

dNK subtypes differentially express interaction partners for proteins expressed by EVT and dESF cells. However, these interactions are difficult to study using primary cells due to limitations in access and cell yield. We have previously generated maternal dESF and placental EVT from iPSC and here established a protocol for differentiation of dNK from iPSC.^35,52^ Our model overcomes limitations of working with primary cells and offers advantages for mechanistic studies using gene editing technologies.^40,41^ In our NT and TGFβ-treated iPSC-derived dNK, we observed expression of *CCL5* and *XCL1*, known partners of *CCR1* and *XCR1*, respectively, expressed by EVT, macrophages, and dendritic cells.^21^ Furthermore, we can induce specific receptors and ligands by modulating the expansion phase. Specifically, NT iPSC-dNK showed high *XCL2* and *KLRB1*, known partners for *XCR1* and *CLEC2D*, respectively, expressed by EVT, dESF, and dendritic cells; conversely, TGFβ-treated iPSC-dNK showed high *CXCR4* and *CSF1*, known partners for *CXCL12* and *CSFR1*, respectively, expressed by dESF and macrophages.^21^ The abundant cell yield and control of receptor expression enables studies of specific interactions affecting placentation and pregnancy outcomes between dNK and other cell types.

A limitation of this study is that we cannot rule out transcriptional similarity to trNK of other tissues. Indeed, this would be an interesting line of investigation, as dNK share expression of CD69 and CD103 with trNK cells found in the lungs.^26,53^ scRNA-seq of human blood, tonsils, lung, and intestinal mucosa clustered NK cells into 4 broad categories: CD56^Bright^ and CD56^Dim^, which can be found in tissues and blood, and PRDM1^+^ ILC1 and ZNF683^+^ NK, found only in tissue. ^54,55^ The authors compared these NK subtypes to dNK and found dNK1 was most similar to intestinal intraepithelial PRDM1^+^ ILC1, while dNK2 and dNK3 most closely resembled ZNF683^+^ NK found in lung and tonsil. We identified *ZNF683* as a dNK2 marker which can be enriched for in iPSC-dNK by TGFβ. These data suggest that follow up analyses of TGFβ-treated iPSC-dNK may find resemblance to lung and tonsil ZNF683^+^ NK– broadening the applications of our protocol.

Just as iPSC-NK are being developed as cellular therapies for cancer treatment, it is paramount that we also develop these technologies into therapeutics for reproductive conditions.

## Materials and Methods

For detailed information and protocols, see Supplementary Materials and Methods

### Patient recruitment and tissue collection

Human decidual and blood tissue samples were collected under UCSD IRB-approved protocols (IRB #181917 and 172111); all patients gave informed consent.

### Primary dNK preparation

First trimester decidual fragments were digested, filtered, and purified by Percoll. Cells were washed, stained, and analyzed by flow cytometry or purified further by NK Cell MACS (Miltenyi Biotec) for functional analyses.

Term placental basal plate was dissected from a 2-3mm depth of the maternal surface. The maternal surface of the chorioaminotic membranes was scraped to isolate cells. All tissues were digested, filtered, and purified by Percoll. Cells were washed, stained, and analyzed by flow cytometry or purified further by FACS using an Aria II sorter (CD3^−^CD19^−^CD15^−^CD45^+^CD56^+^) for functional analyses.

### Peripheral Blood Mononuclear Cell sample collection

PBMCs were isolated from the buffy coat layer of human blood samples. Cells were washed, stained, and analyzed by flow cytometry or purified further by NK Cell MACS (Miltenyi Biotec) for functional analyses.

### hPSC differentiation to dNK-like cells:

Human pluripotent stem cell (hPSC) experiments were performed under a protocol approved by the UCSD Institutional Review Board and Embryonic Stem Cell Research Oversight Committee. Human embryonic stem cell (hESC) WA09/H9 and 3 iPSC lines (established as a part of the Center for Perinatal Discovery) were used for differentiation. After 3 weeks of expansion with or without TGFβ, iPSC-NK were stimulated with PMA/I.

### Flow Cytometry

Single cell suspensions were collected in FACS Buffer (10% FBS, 5mM EDTA in PBS) for antibody staining (Supplementary Table 4) and analyzed using BD FACS Canto (cell surface) or BD Fortessa X20 (intracellular).

### Proteomic profiling

Aptamer-based proteomic profiling was performed using the SomaScan Assay V4.1 (SomaLogic, Boulder, CO, USA) 7K panel.

### Single cell RNA-sequencing (scRNA-seq) reanalysis

Vento-Tormo, 2019 dataset containing first trimester and peripheral blood cells was obtained from cellxgene. Raw data associated with placental villous (PV), basal plate (BP), and chorioamniotic membrane (CAM) tissues from Pique-Regi, 2019 (Version 1) were downloaded with permission from **dbGaP Study Accession:** phs001886.v3.p1.

### scRNA-seq library generation and analysis of iPSC-dNK

Cells differentiated from two iPSC lines with or without TGFβ, were run on the 10X Genomics platform with the Chromium Next GEM Single Cell 3’ v3 kit.

### Statistical Tests

Statistical analysis was performed using GraphPad Prism 10 and R.

## Supplementary Material

Supplement 1

Supplement 2

Supplement 3

Supplement 4

Supplement 5

Supplement 6

Supplement 7

Supplement 8

Supplement 9

Supplement 10

Supplement 11

Supplement 12

## Figures and Tables

**Table 1.** Top 25 marker genes distinguishing dNK cell subtypes (dNK1, dNK2, dNK3, dNKp) and PB-NK cell subtypes (PB-NK CD16^+^, PB-NK CD16^−^)

dNK1	dNK2	dNK3	dNKp	PB-NK CD16-	PB-NK CD16+
B4GALNT1	CDHR1	ITM2C	PBK	IL7R	FGFBP2
CYP26A1	CD2	CXCR4	CENPA	SELL	S1PR5
ENTPD1	ZNF683	TAGAP	CDCA3	IL18R1	AKR1C3
SPINK2	COX6A2	CD160	PLK1	IGFBP4	SPON2
TPTEP1	TNFRSF4	LINC01871	DLGAP5	RIPOR2	PRSS23
C15orf48	KRT81	RGCC	KIF23	LTB	FCGR3A
EPAS1	CTSA	PDE4B	UBE2C	PLAC8	ADGRG1
CDKN1A	IFI44L	DUSP4	PIMREG	TXNIP	PLAC8
CSF1	C1orf162	ARL4A	CCNA2	LINC00861	S100A8
UBASH3B	IGFBP1	RGS2	HMMR	RASGRP2	LINC00861
UBE2F	ITGAX	DNAJB1	SPC25	GZMK	TBX21
DAPK2	BCO2	CEMIP2	TOP2A	IGKC	RIPOR2
ACY3	SNRNP25	RASGEF1B	CEP55	TCF7	LYAR
SYNGR1	KIR2DL4	ZNF331	RRM2	MCUB	GNG2
ID3	IGFBP2	IFRD1	AURKB	KLF2	CD160
PLPP1	MATK	NFE2L2	CKAP2L	GRK6	TXNIP
MDM2	TSPO	ZEB2	CDCA5	KLRF1	CLEC2D
STAT3	AGTRAP	CCNH	CDK1	SNHG25	S100A9
CD59	TESC	PIK3R1	CCNB2	LINC01871	C1orf21
SLC12A8	SLC44A1	PTPN22	SPC24	ITGAM	ICAM2
AFAP1L2	LASP1	PMAIP1	KIF2C	S100A9	TTC38
PIK3R6	AHNAK	ALOX5AP	GTSE1	TRGC2	FAM107B
CAMK1	SEPTIN11	TIPARP	CDCA8	ATM	C12orf75
BCL2L11	GZMH	SERTAD1	NUSAP1	ARHGAP15	LBH
SRGAP3	CHCHD10	ITGAE	BIRC5	VPS51	PPP2R5C

## Significance Statement:

Alterations in maternal decidual natural killer cells (dNK) are associated with pregnancy complications – from recurrent pregnancy loss to preeclampsia and preterm birth. We found dNK from different regions of the term placenta to be distinct from peripheral blood NK and early gestation dNK, based on gene and surface marker expression, subtype composition, and secretome. We report a novel, reproducible protocol to generate dNK resembling the most abundant dNK subtype in early gestation from induced pluripotent stem cells. Our study lays the foundation for *in vitro* modeling of the maternal-fetal interface and therapeutic development for reproductive disease.
