# Supplementary material for: Derivation of functional early gestation decidual natural killer cell subtypes from induced pluripotent stem cells
Source: bioRxiv. 2025 Nov 5:2025.11.03.685424. Preprint. [Version 1] doi: 10.1101/2025.11.03.685424 (PMC12637421; doi:10.1101/2025.11.03.685424)
Supplement: Supplement 2 [file media-2.pdf]

Supplementary Table 1. Pathway analysis of NT clade using Descartes_Cell_Types_and_Tissue_2021 library

Term	# of Enriched Genes	# Term Genes	Adjusted P-value
Microglia in Cerebellum	122	606	3.40E-14
Lymphoid cells in Intestine	48	156	3.23E-12
Microglia in Eye	56	240	4.71E-09
Lymphoid cells in Placenta	39	153	1.67E-07
Lymphoid cells in Adrenal	40	177	3.14E-06
Lymphoid cells in Stomach	23	85	4.33E-05
Microglia in Cerebrum	75	465	4.33E-05
Lymphoid cells in Lung	33	150	4.51E-05
Lymphoid cells in Heart	24	110	9.94E-04
Lymphoid cells in Pancreas	29	152	0.00217016

Supplementary Table 2. Pathway analysis of TGFβ clade using Descartes_Cell_Types_and_Tissue_2021 library.

Term	# of Enriched Genes	# Term Genes	Adjusted P-value
Lymphoid cells in Intestine	22	156	2.21E-06
Lymphoid cells in Placenta	20	153	1.65E-05
Lymphoid cells in Adrenal	17	177	0.00445743
Lymphoid cells in Lung	13	150	0.04792624
Microglia in Eye	17	240	0.06963168
Lymphoid cells in Heart	10	110	0.06963168
Lymphoid cells in Spleen	10	119	0.09212431
Lymphoid cells in Stomach	8	85	0.09212431
Lymphoid cells in Kidney	17	260	0.09212431
Lymphoid cells in Muscle	13	193	0.14822724

Supplementary Table 3. Pathway analysis of proliferative clade using Descartes_Cell_Types_and_Tissue_2021 library

Term	# of Enriched Genes	# Term Genes	Adjusted P-value
Erythroblasts in Stomach	8	115	9.47E-05
Erythroblasts in Heart	10	331	0.00414751
Erythroblasts in Muscle	5	191	0.11891546
Megakaryocytes in Muscle	4	288	0.86249075
Erythroblasts in Pancreas	2	140	0.93551307
Lymphoid cells in Placenta	2	153	0.93551307
Trophoblast giant cells in Placenta	1	89	0.99433571
Lens fibre cells in Eye	1	111	0.99433571
Smooth muscle cells in Eye	1	135	0.99433571
Megakaryocytes in Lung	1	191	0.99433571

Supplementary Table 4. List of antibodies used for flow cytometry

Protein	Fluorochrome	Clone	Vendor	Catalog#
CD19 (Lineage)	PerCP-Cy5.5	HIB19	Biolegend	302230
CD15 (Lineage)	PerCP-Cy5.5	W6D3	Biolegend	323020
CD3 (Lneage)	PerCP-Cy5.5	OKT3	Biolegend	317335
7-AAD			ThermoFisher	A1310
CD45	APC-Cy7	HI30	Biolegend	304014
CD45	BV650	HI30	Biolegend	304044
CD45	APC	HI30	BD	560973
CD56	APC	B159	BD	555518
CD56	PE-Cy7	MEM-188	Biolegend	304628
CD16	FITC	3G8	Biolegend	302006
CD18	PE-Cy7	1B4	Biolegend	373410
KIR2DL1	PE	1127B	R&D Systems	FAB8887P-025
CD9	PE	HI9a	Biolegend	312106
CD103	PE-Cy7	Ber-ACT8	Biolegend	350212
CD39	PE	A1	Biolegend	328208
CD69	PE-Cy7	FN50	Biolegend	310912
CD49a	AlexaFlour 647	TS2/7	Biolegend	328310
CD34	APC	581	BD	555824
CD34	PE-Cy7	581	BD	560710
SSEA4	PE	MC-813-70	Biolegend	330406
CD107a	BV510	H4A3	Biolegend	328632
IFNγ	APC/Cy7	B27	Biolegend	506524
TNFα	PE/Cy7	MAb11	Biolegend	502930
GM-CSF	Pe/dazzle™ 594	BVD2-21C11	Biolegend	502318
PFN	BV711	dG9	Biolegend	308129
GNLY 9kD	PE	DH2	Biolegend	348004
VEGF	APC	23410	R&D Systems	IC2931A
Isotype Controls				
Brilliant Violet 510™ Mouse IgG1, κ Isotype Ctrl Antibody	BV510	MOPC 21	Biolegend	400172
APC/Cyanine7 Mouse IgG1, κ Isotype Ctrl Antibody	APC-Cy7	MOPC 21	Biolegend	400128
PE/Cyanine7 Mouse IgG1, κ Isotype Ctrl Antibody	PE-Cy7	MOPC 21	Biolegend	400126

PE/Dazzle™ 594 Rat IgG2a, κ Isotype Ctrl Antibody	Pe/dazzle™ 594	RTK2758	Biolegend	400558
Brilliant Violet 711™ Mouse IgG1, κ Isotype Ctrl Antibody	BV711	MOPC 21	Biolegend	400168
PE Mouse IgG1, κ Isotype Ctrl	PE	MOPC 21	Biolegend	400112
Anti IgG1 κ Mouse, APC	APC	MOPC 21	Biolegend	400120
