# Supplementary figures and images for: Derivation of functional early gestation decidual natural killer cell subtypes from induced pluripotent stem cells
Source: bioRxiv. 2025 Nov 5:2025.11.03.685424. Preprint. [Version 1] doi: 10.1101/2025.11.03.685424 (PMC12637421; doi:10.1101/2025.11.03.685424)

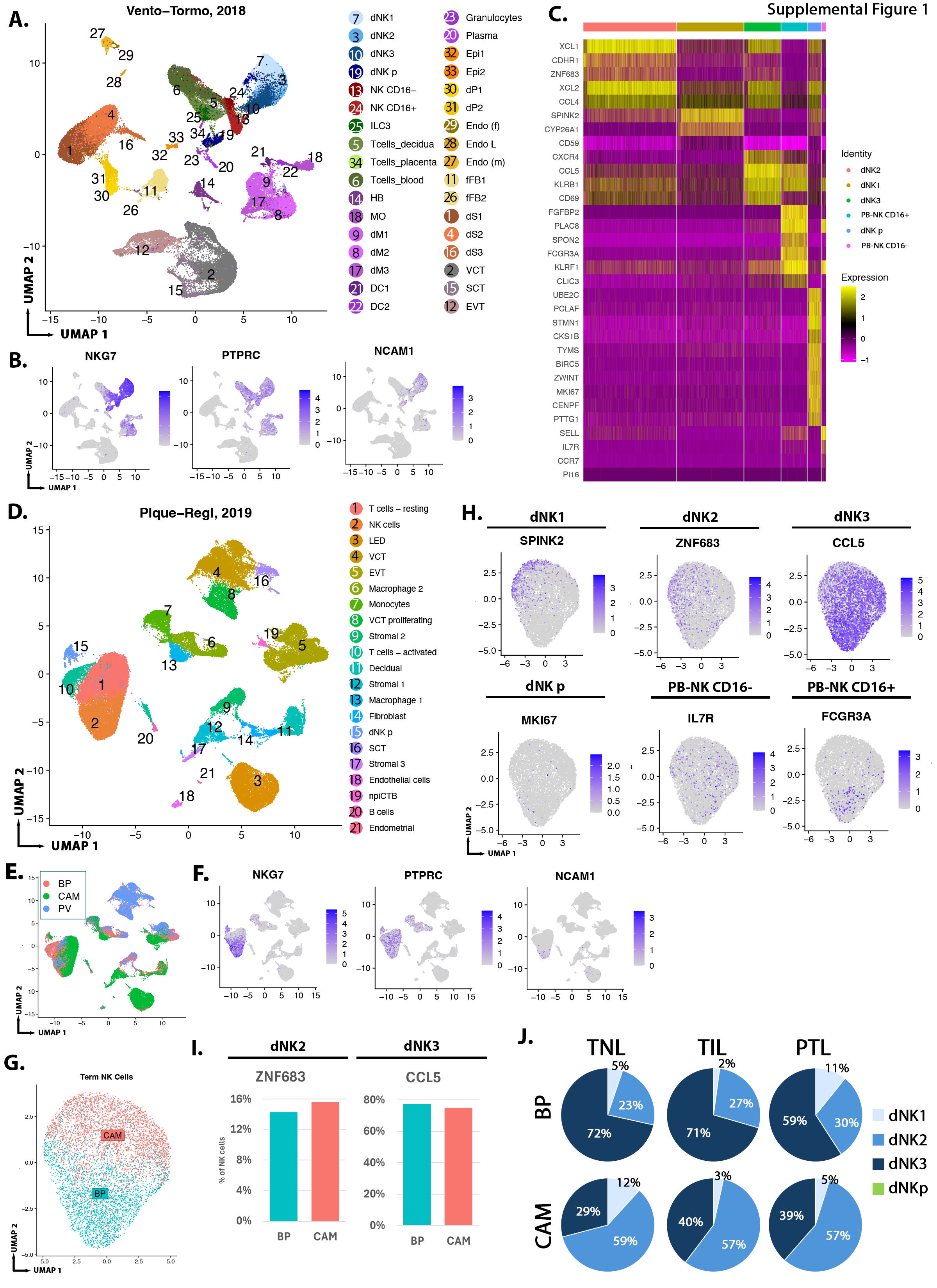

Supplement: Supplement 7 [file media-7.jpg]

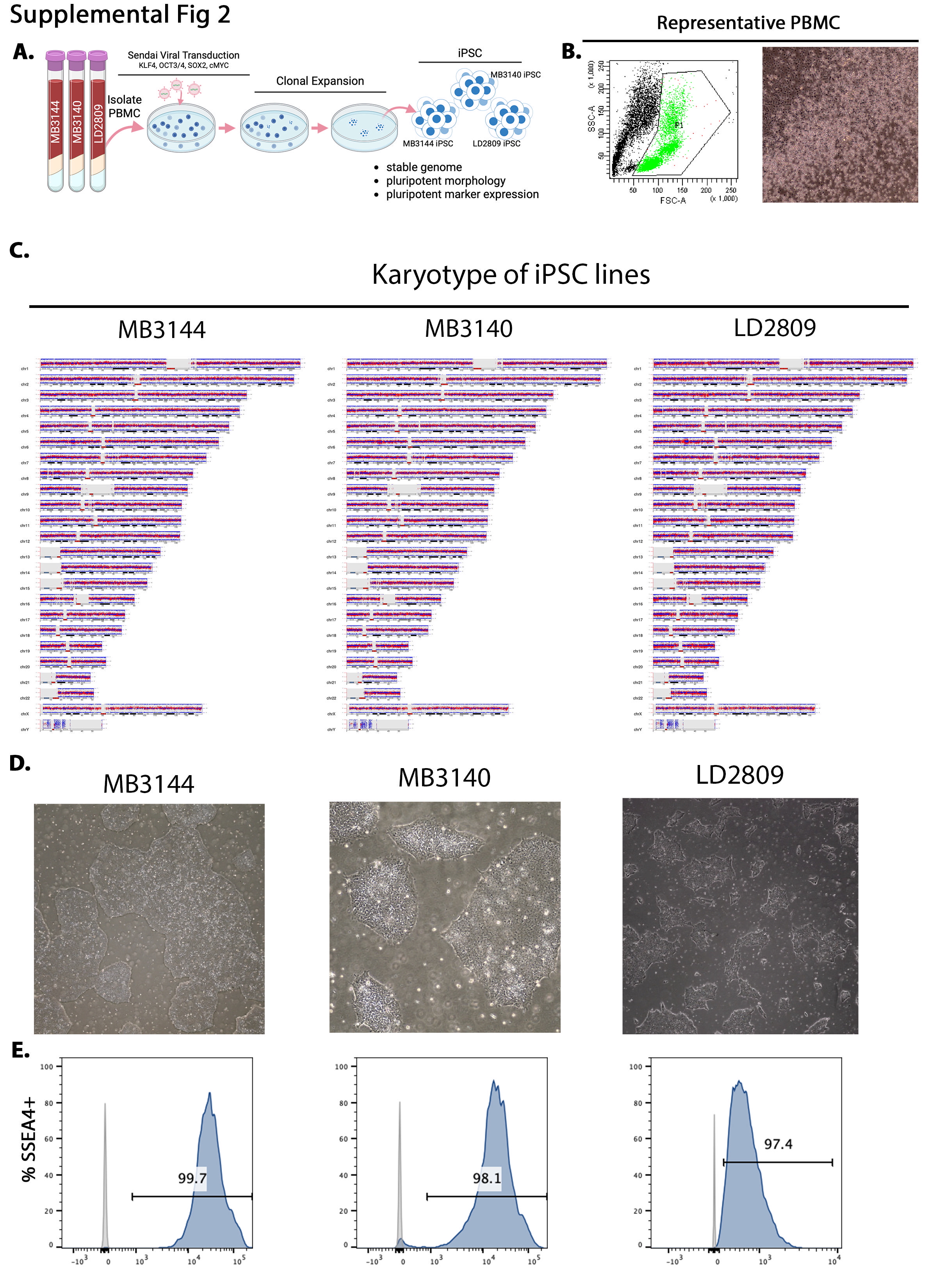

Supplement: Supplement 8 [file media-8.jpg]

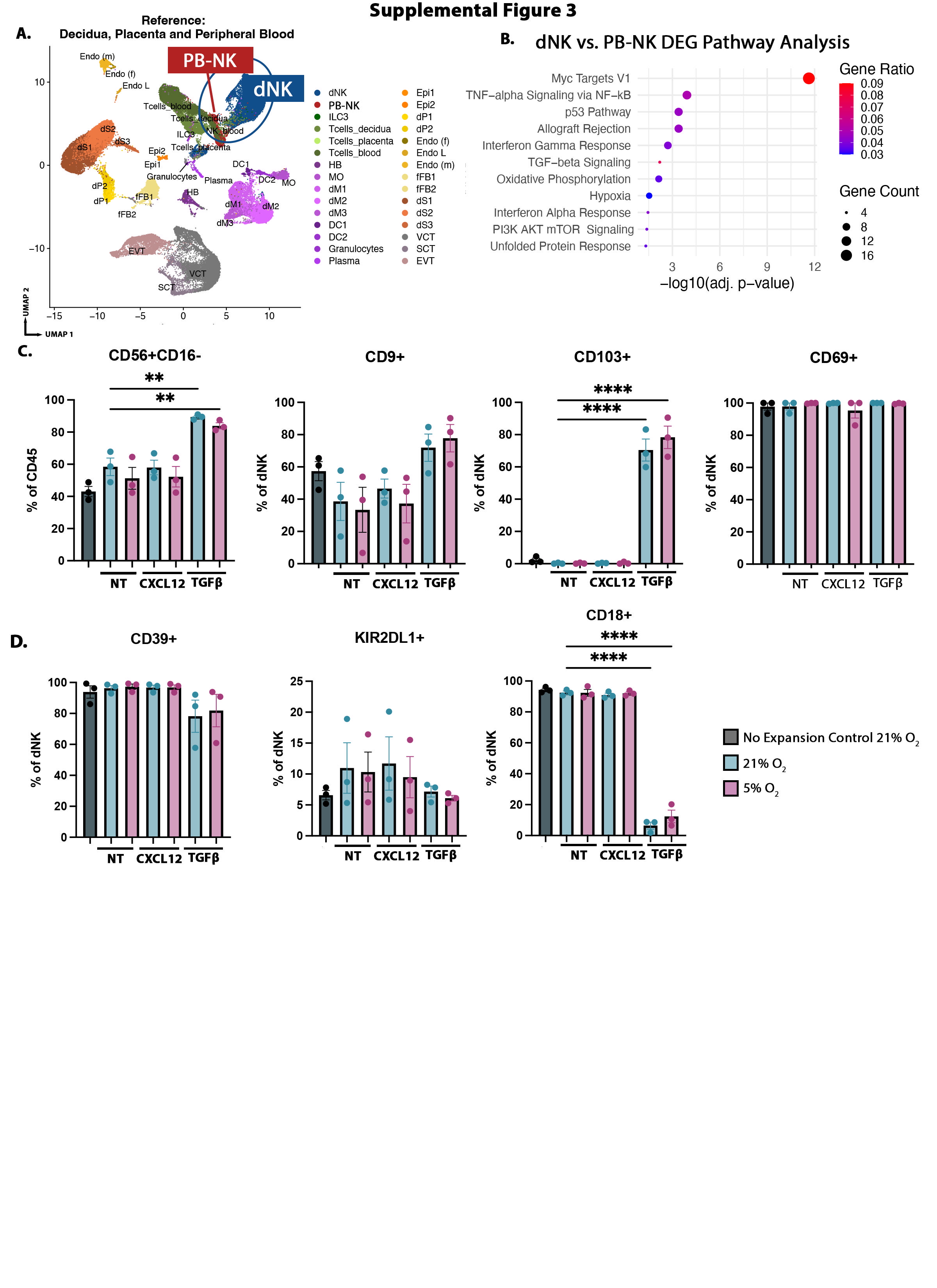

Supplement: Supplement 9 [file media-9.jpg]

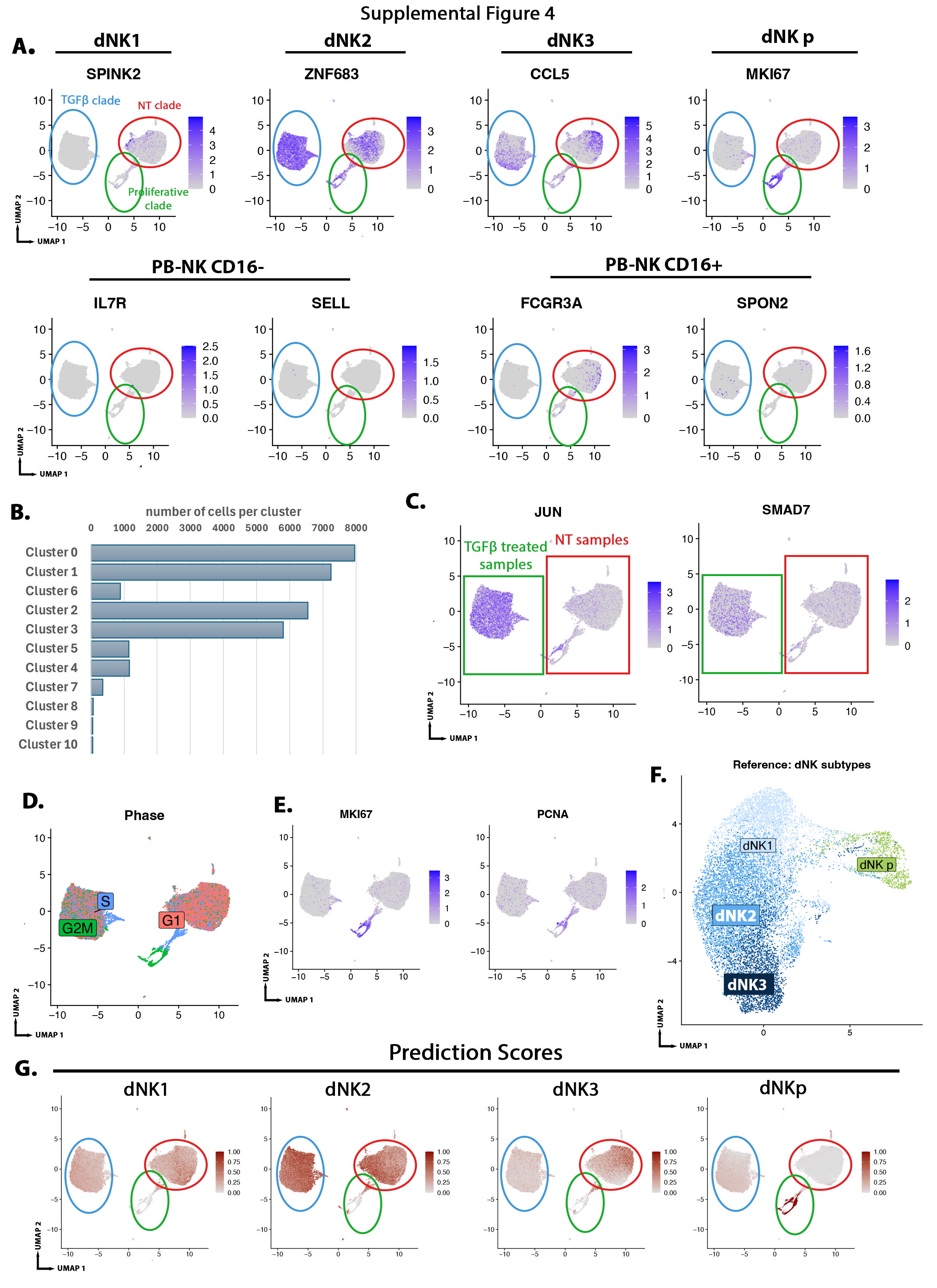

Supplement: Supplement 10 [file media-10.jpg]

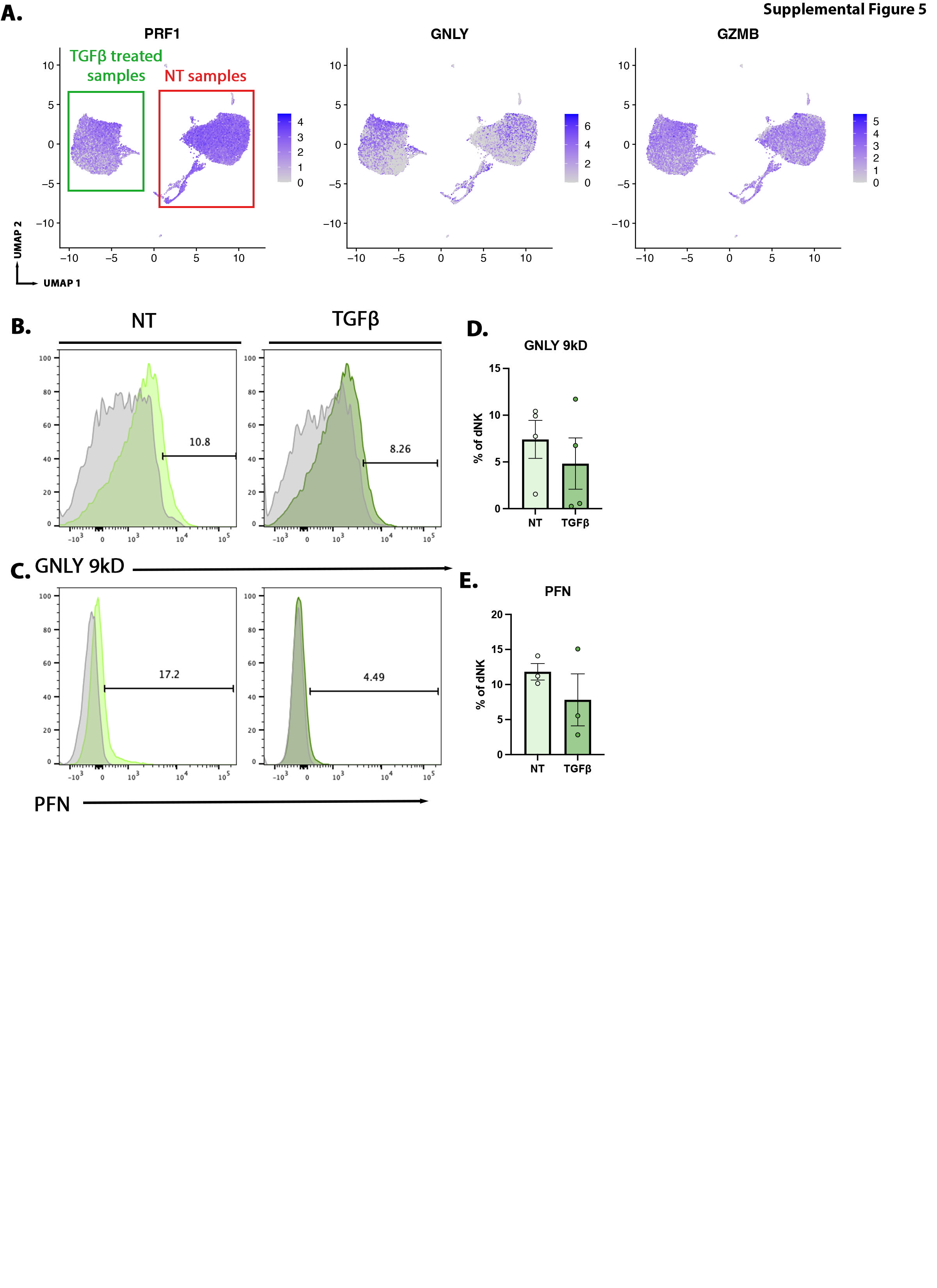

Supplement: Supplement 11 [file media-11.jpg]

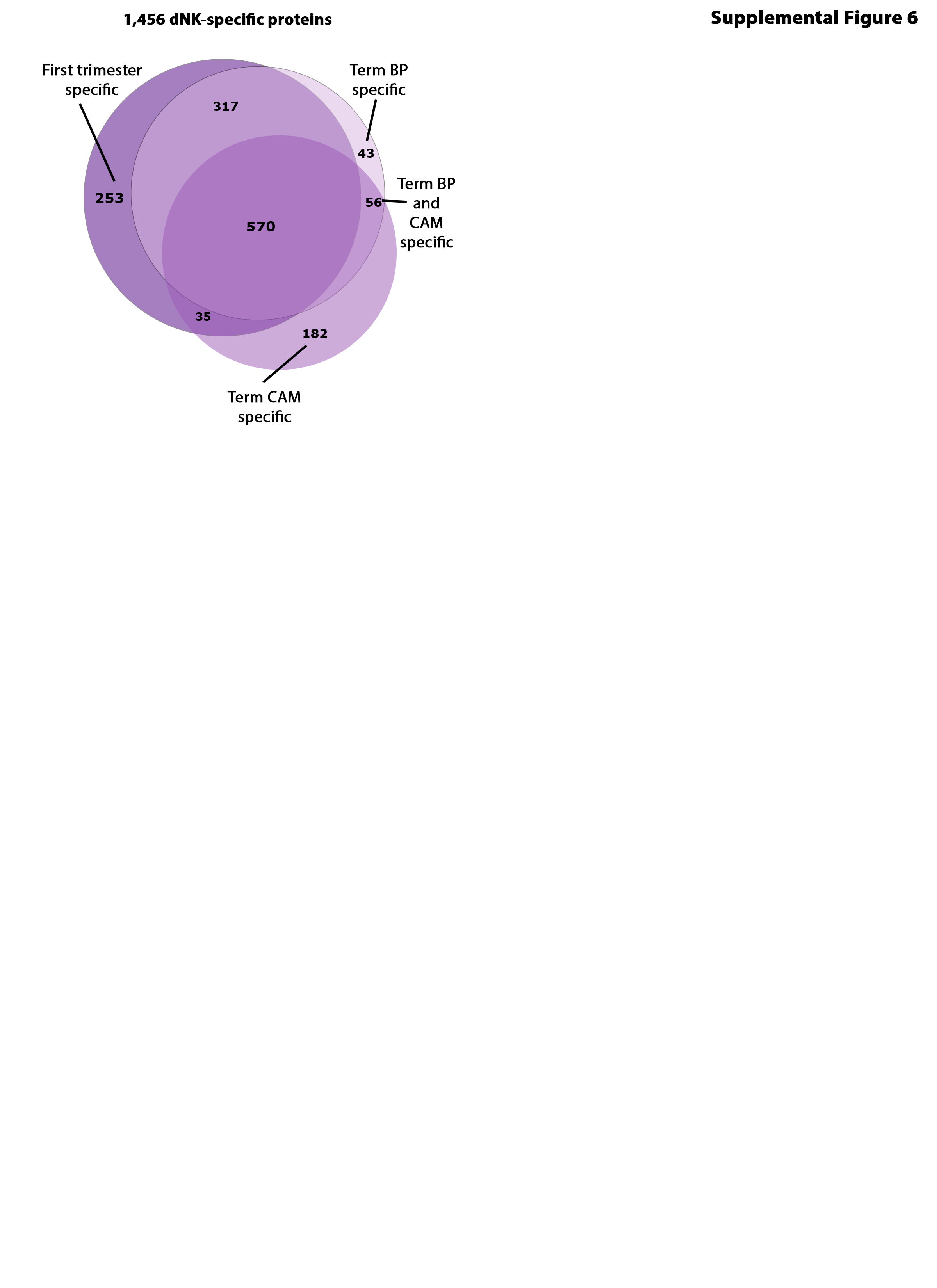

Supplement: Supplement 12 [file media-12.jpg]
